# Structural valve deterioration of the Labcor Dokimos aortic prosthesis: a single-centre experience

**DOI:** 10.1093/icvts/ivab286

**Published:** 2021-10-23

**Authors:** Dounia Iskandarani, Omar Chaabo, Walid Gharzeddine, Pierre Sfeir, Mounir Obeid, Ziyad Ghazzal, Abdallah Rebeiz, Fadi J Sawaya

**Affiliations:** Division of Cardiology, American University of Beirut Medical Center, Beirut, Lebanon; Division of Cardiology, American University of Beirut Medical Center, Beirut, Lebanon; Division of Cardiology, American University of Beirut Medical Center, Beirut, Lebanon; Division of Cardiology, American University of Beirut Medical Center, Beirut, Lebanon; Division of Cardiology, American University of Beirut Medical Center, Beirut, Lebanon; Division of Cardiology, American University of Beirut Medical Center, Beirut, Lebanon; Division of Cardiology, American University of Beirut Medical Center, Beirut, Lebanon; Division of Cardiology, American University of Beirut Medical Center, Beirut, Lebanon

**Keywords:** Transcatheter valve therapy, Valve disease, Education cardiac

## Abstract

**OBJECTIVES:**

The goal of this study was to assess the performance and incidence of the deterioration of the Labcor Dokimos bioprosthetic aortic valve.

**METHODS:**

We performed a retrospective medical chart review of 116 patients who underwent surgical aortic valve replacement with the Labcor Dokimos aortic valve between 2010 and 2018. Abstracted data included patient demographic and echocardiographic data. Patients were divided into 2 groups: patients with structural valve deterioration (SVD) and patients without SVD.

**RESULTS:**

Among the patients with complete follow-up (*n* = 95), 10 patients were excluded because they died within a year; 85 patients were included in the final analysis. Of the 85 patients, 32 (38%) developed SVD; 22 (26%) had severe SVD, 15 (18%) of whom underwent reintervention. The most common aetiology of SVD was severe central aortic regurgitation, which was detected in 91% of the patients who had severe SVD. The average time from operation to severe SVD was 4.7 years with a minimum of 1.5 years and a maximum of 7.9 years.

**CONCLUSIONS:**

Bioprosthetic aortic valve deterioration due to severe aortic regurgitation is common and occurs early with the Labcor Dokimos valve. This occurrence needs to be furthered investigated in larger registries.

**Figure:**
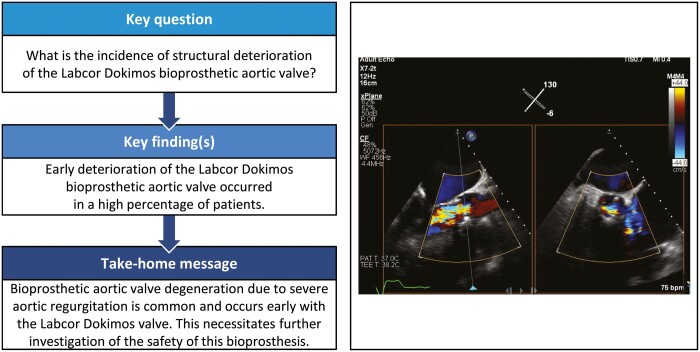

## INTRODUCTION

The advent of less invasive therapies, valve-in-valve transcatheter aortic valve implantation (TAVI) for treating bioprosthetic valve failure and patient preference against chronic anticoagulation therapy has driven a major increase in the use of aortic bioprostheses in younger patients. According to the most recent American College of Cardiology/American Heart Association guidelines, bioprosthetic valves are now considered in younger patients from 50 to 70 years old [1]. According to the European Society of Cardiology, patients who are 60 years and older could be treated with a bioprosthetic valve [2].

Bioprosthetic tissue used in such valves undergoes a time-dependent process of structural changes, resulting in dysfunction commonly beginning after 8 years after implantation and necessitating the replacement of the valve after 10–15 years [3, 4].

Given this increasing prevalence of bioprosthetic heart valves in younger patients, new valves should be studied rigorously before widespread dissemination to avoid similar scenarios of early structural valve deterioration (SVD) like the one seen with the Mitroflow valve (Sorin Group, Inc., Milan, Italy) [5].

The goal of this single-centre, retrospective study was to investigate the performance and incidence of SVD of the Labcor Dokimos bioprosthetic aortic valve (Labcor, Belo Horizonte, Brazil).

## PATIENTS AND METHODS

### Data collection

We performed a retrospective search of patient information from January 2010 through December 2018. Data from all consecutive patients with aortic valve diseases who underwent surgical aortic valve replacement (SAVR) with the Labcor valve with or without concomitant procedures in our department were analysed.

Data from a total of 116 patients were retrieved. All these patients had transthoracic echocardiograms (TTE) performed postoperatively by our department.

### Ethical statement

The institutional review board granted approval for this study, and informed consent was waived.

### Labcor Dokimos bioprosthesis

The Labcor Dokimos prosthesis is a CE-marked stented bovine pericardial supra-annular bioprosthesis, available in sizes from 19 to 27 mm. Special features include its low-profile stent and ample suture cuff, externally mounted leaflets that allow for optimum blood flow and a premolded tissue-to-tissue interface that minimizes the risk of leaflet abrasion and SVD [6]. The Labcor Dokimos Plus also features a ‘reducer’ treatment that claims to reduce antigenicity and lipid content as well as major binding calcium sites, hence reducing calcification. The ‘reducer’ treatment does not have clinical data that evaluate the long-term impact in patients.

### Echocardiographic measurements

All patients were routinely evaluated postoperatively and annually using TTE performed according to the guidelines of the European Association of Cardiovascular Imaging and the American Society of Echocardiography. The TTE reports included left ventricular function involving left ventricular outflow tract (LVOT) flow velocity and LVOT diameter. TTE also included aortic valve function that involved paravalvular leakage, aortic regurgitation (AR), pressure gradients (peak and mean) and indexed effective orifice area (EOAi), acceleration time and Doppler velocity indices (DVI).

Patient–prosthesis mismatch (PPM) was also calculated in all patients and defined as moderate PPM ≥ 0.6–0.85 cm^2^/m^2^ and severe PPM ≤ 0.6 cm^2^/m^2^ [7].

### Definition of structural valve deterioration

According to the European Society of Percutaneous Cardiovascular Interventions and the endorsement of the European Society of Cardiology, moderate SVD is defined as (i) mean gradient ≥20 and <40 mmHg and/or ≥10 and <20 mmHg change from baseline (before discharge or within 30 days of valve implantation) and/or (ii) moderate or new worsening (>1+/4+) of intraprosthetic AR. Severe SVD is defined as a mean gradient of ≥40 mmHg or an increase in the mean gradient of ≥20 mmHg from baseline or severe intraprosthetic AR, new or worsening (>2/4) from baseline [8].

### Surgical technique

SAVR was performed via a median sternotomy using standard cardiopulmonary bypass with mild hypothermia and cold crystalloid cardioplegia. The aorta was clamped, and cold custodial hypothermic cardioplegia solution was infused into the aortic root until total cardiac standstill was obtained. A transverse aortotomy was performed, and exposure to the aortic valve was gained. The aortic valve was excised and decalcified. Prosthesis sizing was determined via sizers provided by the manufacturer. The valve was sutured in the aortic position using interrupted pledgeted sutures.

### Statistics

All data were retrospectively collected and analysed with SPSS Statistics version 22.0.0 (SPSS Inc., Chicago, IL, USA). Univariate descriptive statistical analyses were performed to describe the measures of central tendency and the distributions of the variables. Continuous variables are expressed as mean and standard deviation. Categorical variables are presented as proportion and absolute number. Differences between patients who developed SVD and patients who did not develop SVD were detected using the χ^2^ test or the Fisher exact test for categorical variables and the Student's t-test or the Mann–Whitney *U*-test for continuous variables. Multiple logistic regression analysis was performed to study the predictors of SVD of the Labcor Dokimos valve; Cox regression analysis was used to study the impact of selected variables on SVD.

## RESULTS

Out of 116 patients, a total of 21 patients were lost to follow-up because they resided outside Lebanon. Of the remaining 95, 10 patients died either intra- or postoperatively or within 1 year from the operation. A total of 85 patients were included in the analysis.

###  

#### Patient demographics

Patient demographic data and clinical characteristics for patients who developed SVD and those who did not are shown in Table 1. There was no statistical difference between the 2 groups. Patients in both groups were septuagenarians, with a mean age of 73.8 and 71.9 years in the non-SVD and SVD groups, respectively. Patients in both groups were predominantly men and had severe aortic stenosis (AS) as the main valvular disease. Within the first year, 10 patients had died; of those, 8 patients died either intraoperatively due to haemodynamic instability or postoperatively during their hospital stay. The causes of death of patients who were excluded from the statistical analysis are included in Supplementary Material, Table S1.

**Table 1:** Baseline characteristics of the population of patients with Labcor Dokimos valves who underwent surgical aortic valve replacement

Clinical data	Without SVD	With known SVD	*P*-value
	*N* = 53	*N* = 32	
Male gender, *n* (%)	29 (54.7)	20(62.5)	0.482
Age (years), mean ± SD	73.8 ± 6.9	71.9 ± 7.23	0.241
BSA (m^2^), mean ± SD	1.81 ± 0.21	1.89 ± 0.21	0.110
STS score (%), mean ± SD	2.14 ± 1.06	2.08 ± 1.09	0.784
Hypertension (%), *n* (%)	49 (92.5)	30 (93.8)	1.000
Diabetes mellitus 2 (%), *n* (%)	13 (24.5)	10 (31.3)	0.499
Dyslipidaemia (%), *n* (%)	21 (39.6)	17 (53.1)	0.225
COPD (%), *n* (%)	4 (7.5)	1 (3.1)	0.646
PAD (%), *n* (%)	3 (5.7)	1 (3.1)	1.000
Stroke (%), *n* (%)	1 (1.9)	0	1.000
CKD (%), *n* (%)	13 (24.5)	8 (25)	0.961
Haemodialysis (%), *n* (%)	1 (1.9)	0	1.000
Atrial fibrillation (%), *n* (%)	5 (9.4)	8 (25)	0.067
Creatinine (mg/dl), mean ± SD	1.23 ± 0.99	1.06 ± 0.34	0.360
Calcium, mean ± SD	9.31 ± 0.51	9.26 ± 0.48	0.651
Cardiac, *n* (%)			
CABG	4 (7.5)	3 (9.4)	1.000
SAVR	5 (9.4)	3 (9.4)	1.000
SMVR	0	1 (3.1)	0.376
PCI	4 (7.5)	4 (12.5)	0.468
Baseline echocardiography			
Valve disease, *n* (%)			0.840
AS	34 (65.4)	20 (64.5)	
AR	11 (21.2)	8 (25.8)	
Mixed	7 (13.5)	3 (9.7)	
LVEF (<50%)	4 (8.2)	7 (22.6)	0.097
AVA (cm^2^), mean ± SD	0.81 ± 0.22	0.85 ± 0.25	0.462
PG (mmHg), mean ± SD	77.47 ± 27.69	77.30 ± 25.65	0.980
MG (mmHg), mean ± SD	47.37 ± 18.79	45.92 ± 17.10	0.749
Medication, *n* (%)			
Statin	30 (57.7)	19 (59.4)	1.000
Antiplatelet	40 (76.9)	22 (68.8)	0.450
Anticoagulation	8 (15)	10 (31.2)	0.795

AS: aortic stenosis; AR: aortic regurgitation; AVA: aortic valve area; BSA: body surface area; CABG: coronary artery bypass surgery; CKD: chronic kidney disease; COPD: chronic obstructive pulmonary disease; LVEF: left ventricular ejection fraction; MG: mean gradient; PAD: peripheral artery disease; PCI: percutaneous coronary intervention; PG: peak gradient; SAVR: surgical aortic valve replacement; SMVR: surgical mitral valve replacement; STS: society of thoracic surgery; SVD: structural valve deterioration.

#### Operative data and early clinical outcomes

Operative and postoperative outcomes are shown in Table 2. Postoperative atrial fibrillation and the need for a new permanent pacemaker were similar in the SVD and the non-SVD groups (37.5% vs 30.2; *P* = 0.487 and 12.5% vs 7.7%; *P* = 0.473, respectively).

**Table 2:** Operative and postoperative details of surgical aortic valve replacement patients who had surgical aortic valve replacement with a Labcor Dokimos valve

	Without SVD	With known SVD	*P*-value
	*N* = 53	*N* = 32	
Valve morphology, *n* (%)			
Tricuspid	51 (96.2)	30 (93.8)	0.630
Valve sizes, *n* (%)			0.544
19 mm	1 (1.9)	1 (3.1)	
21 mm	19 (35.8)	10 (31.3)	
23 mm	23 (43.4)	18 (56.3)	
25 mm	10 (18.9)	3 (9.4)	
New onset AF, *n* (%)	16 (30.2)	12 (37.5)	0.487
New pacemaker, *n* (%)	4 (7.7)	4 (12.5)	0.473
Post-PG (mmHg), mean ± SD	24.75 ± 12.34	26.40 ± 11.61	0.594
Post-MG (mmHg), mean ± SD	12.85 ± 6.67	13.44 ± 7.95	0.738
Post-AR, *n* (%)			0.801
None	30 (68.2)	19 (70.4)	
Mild	12 (27.3)	8 (29.6)	
Moderate	2 (4.5)	0	
Moderate to severe			
Severe			
Post-LVOT diameter, mean ± SD	21.22 ± 1.52	21.30 ± 1.29	0.829
Post-LVOT VTI, mean ± SD	22.12 ± 4.39	22.20 ± 4.82	0.946
Post-aortic VTI, mean ± SD	38.87 ± 10.16	46.13 ± 14.70	0.01
Post-EOA, mean ± SD	2.14 ± 0.54	1.78 ± 0.48	0.006
Post-EOAi, mean ± SD	1.16 ± 0.31	0.93 ± 0.25	0.002
Post-DVI, mean ± SD	0.59 ± 0.11	0.50 ± 0.13	0.004
Patient–prosthesis mismatch, *n* (%)			0.043
None	32 (84.2)	17 (56.7)	
Moderate	5 (13.2)	9 (13.3)	
Severe	1 (2.6)	4 (13.3)	

AF: atrial fibrillation; AR: aortic regurgitation; DVI: Doppler velocity index; EOA: effective orifice area; EOAi: indexed effective orifice area; LVOT: left ventricular outflow tract; MG: mean gradient; PG: peak gradient; VTI: velocity time integral; SVD: structural valve deterioration.

Post-effective orifice area (EOA), EOAi and DVI were higher in the non-SVD group (2.14 ± 0.54, 1.16 ± 0.31 and 0.59 ± 0.11, respectively) versus in the SVD group (1.78 ± 0.48, 0.93 ± 0.25 and 0.50 ± 0.13 with *P* < 0.01, respectively). Moderate and severe PPM were more common in the SVD than in the non-SVD group (26.3 vs 15.8%; *P* = 0.043, respectively).

Table 3 shows postoperative gradients, degree of AR, LVOT diameter, LVOT and aortic velocity time integral and orifice areas according to the size of the implanted prosthesis. Severe PPM was observed (15.8%) primarily with size 21 mm.

**Table 3:** Echocardiographic parameters at discharge according to valve size

	19 mm	21 mm	23 mm	25 mm	*P*-value
LVEF (<50%), *n* (%)	1 (50)	2 (10)	5 (14.7)	3 (27.3)	0.293
PG (mmHg), mean ± SD	19.50 ± 12.02	29 ± 15.06	23.53 ± 9.59	25.27 ± 12.10	0.390
MG (mmHg), mean ± SD	11 ± 8.48	14.75 ± 7.33	12.03 ± 7.13	13.36 ± 7.14	0.589
AR, *n* (%)	2	22	36	11	0.592
None	2 (100)	14 (63.6)	25 (69.4)	8 (72.7)	
Mild		6 (27.3)	11(30.6)	3 (27.3)	
Moderate		2 (9.1)			
Severe					
LVOT diameter, mean ± SD	20.5 ± 0.70	20.35 ± 1.18	21.39 ± 1.02	22.7 ± 1.94	0.000
LVOT VTI, mean ± SD	13.5 ± 6.36	23.65 ± 5.07	21.89 ± 3.92	21.90 ± 3.81	0.019
Aortic VTI, mean ± SD	32 ± 16.97	47.9 ± 14.53	39.94 ± 10.02	39.90 ± 15.22	0.076
EOA, mean ± SD	1.5 ± 0.14	1.76 ± 0.52	2.03 ± 0.44	2.32 ± 0.76	0.028
EOAi, mean ± SD	0.94 ± 0.16	0.99 ± 0.32	1.07 ± 0.27	1.17 ± 0.44	0.495
DVI, mean ± SD	0.48 ± 0.09	0.53 ± 0.15	0.57 ± 0.12	0.56 ± 0.13	0.630
Patient–prosthesis mismatch, *n* (%)	2	19	37	10	0.296
None	1 (50)	11 (57.9)	30(81.1)	7 (70)	
Moderate	1 (50)	5 (26.3)	6 (16.2)	2 (20)	
Severe		3 (15.8)	1 (2.7)	1 (10)	

AR: aortic regurgitation; DVI: Doppler velocity index; EOA: effective orifice area; EOAi: indexed effective orifice area; LVEF: left ventricular ejection fraction; LVOT: left ventricular outflow tract; MG: mean gradient; PG: peak gradient; VTI: velocity time integral.

#### Cumulative incidence of structural valve deterioration and reoperation

**Figure 1:**
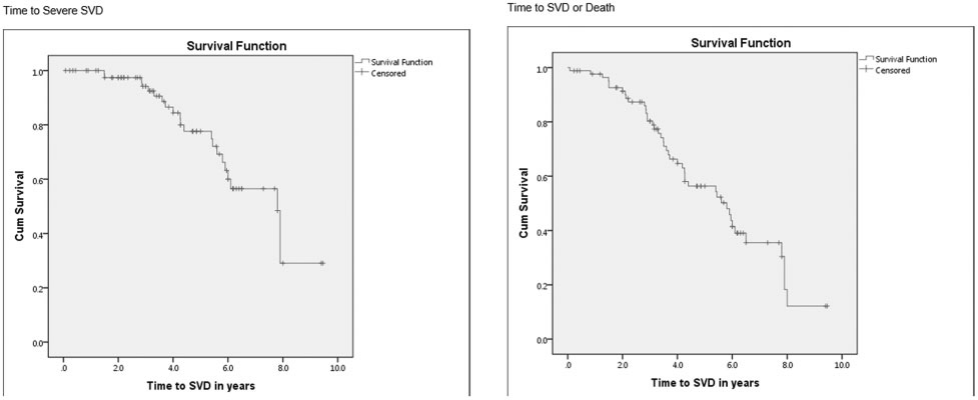
Time to structural valve deterioration—Kaplan–Meier curve. Cum: cumulative; SVD: structural valve deterioration.

The incidence of SVD and reintervention is shown in Supplementary Material, Table S2. Out of 85 patients, 38% developed a degree of SVD. Moderate SVD was found in 12% of the patients whereas severe SVD was found 26% of the patients. The average time from operation to severe SVD was 4.7 years with a minimum of 1.5 years and a maximum of 7.9 years (Fig. 1).

Among patients who developed severe SVD, 68% had undergone reoperation; 27% underwent redo SAVR and 41% had valve-in-valve TAVI. The rest are asymptomatic or awaiting reintervention.

#### Mode of deterioration

**Figure 2:**
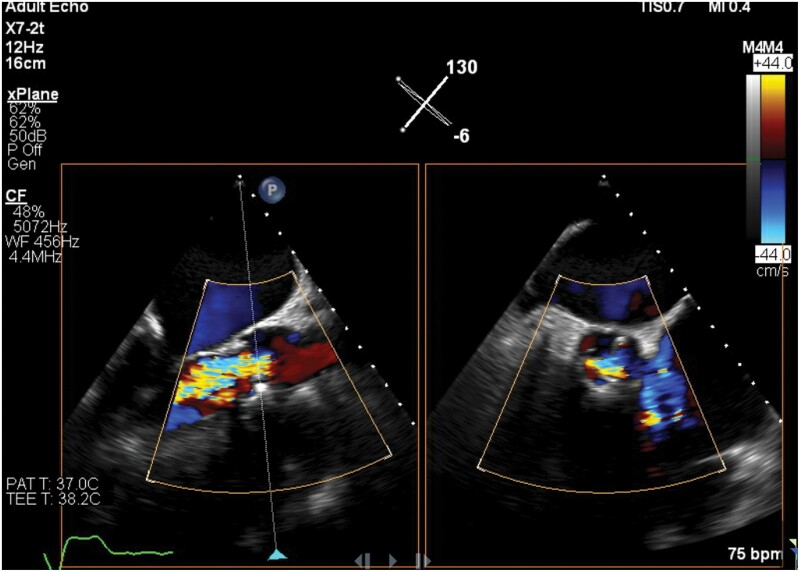
Echocardiographic images of central aortic regurgitation.

**Figure 3:**
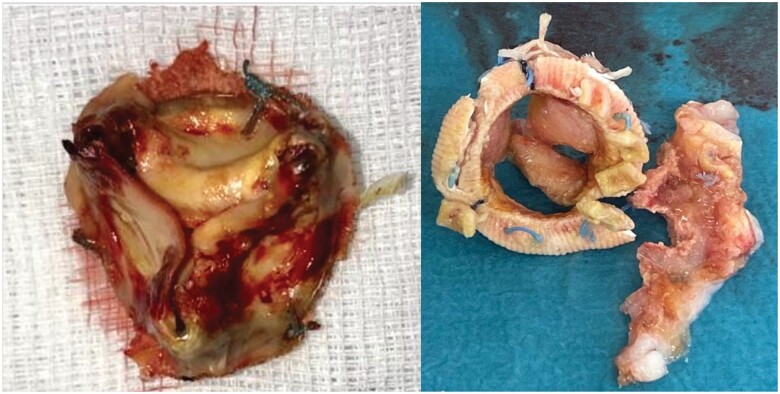
Pannus formation underneath Labcor valve extracted intraoperatively.

During the follow-up period, 22 cases of severe SVD were diagnosed according to echocardiographic criteria. Three modes of SVD were observed: The main mode was AR (91%), whereas AS and mixed AR/AS occurred in only 9% of the patients. Severe AR presented as severe central AR in all the patients on TTE. In cases in whom a TEE study was done, severe central AR was due to [1] leaflet non-coaptation [2]; prolapse of the non-coronary sinus cusp, which appears to show fluttering in diastole suggestive of a fracture leaflet [3]; apparent retraction of the anterior leaflet; and [4] abnormal excursion of 1 leaflet (Fig. 2). In patients who had redo SAVR, intraoperative findings included 2 cases with significant pannus formation underneath the valve and others with lack of coaptation (Fig. 3). Supplementary Material, Table S3 summarizes the mode of deterioration of the Labcor valve according to size.

### Predictors of early structural valve deterioration

A multivariable logistic regression analysis was conducted to investigate the predictors of severe SVD; the results are presented in Table 4. With past literature reviews emphasizing PPM and smaller valve sizes as factors associated with early SVD, the following predictors were chosen: PPM, valve size and post SAVR EOA. These predictors were not found to have a significant impact on early SVD. We constructed a forward stepwise multivariate Cox regression model with severe SVD as the outcome (dependent) variable with age, gender, PPM, valve size, post EOA and chronic kidney disease as independent variables (Table 5). The only variable that had an independent and significant association with severe SVD was post EOA (hazard ratio per 1 cm^2^ increase: 0.383, 95% CI: 0.158–0.928; *P* = 0.034).

**Table 4:** Multivariable logistic regression analysis for the predictors of severe structural valve deterioration

	SVD			*P*-value
	OR	95% CI		
		Lower	Upper	
Patient–prosthesis mismatch (reference: none)	1.920	0.397	9.283	0.417
Post effective orifice area	0.305	0.070	1.325	0.113
Valve size (reference: 23 mm)	0.590	0.197	1.764	0.345

CI: confidence interval; OR: odds ratio; SVD: structural valve deterioration.

**Table 5:** Cox analysis for severe structural valve deterioration

Variables in the Equation									
		*B*	SE	Wald	df	Sig.	Exp(*B*)	95.0% CI for Exp(*B*)	
								Lower	Upper
Step 1	Post_EOA	−0.960	0.451	4.519	1	0.034	0.383	0.158	0.928

CI: confidence interval; EOA: effective orifice area.

Similarly, a forward stepwise multivariate Cox regression model was done with the same independent variables but having SVD or death as the outcomes. None of the variables had an independent and significant association.

## DISCUSSION

Our report shows that severe structural aortic valve degeneration with the Labcor Dokimos valve is [1] common and occurred in 26% of the cases [2]; occurs early, with time to severe SVD of 4.7 years [3]; has severe central AR as the mode of failure in 91% cases; and [4] has a larger EOA post SAVR that is protective against SVD.

Prior studies assessed the performance of the Labcor Dokimos aortic valve postoperatively without short- or long-term follow-up [9, 10]. In a single-centre study assessing the performance of the bioprosthesis, the haemodynamic results were satisfactory, i.e. echocardiographic parameters such as reduction in pressure gradients and increase in EOAi improved significantly [9]. In that study, patients were only followed up postoperatively and had a TTE within 10 days of implantation. Out of 137 patients, 4 had moderate to severe paravalvular leakage that required reintervention. Another study that included 100 patients showed similar results in which no relevant central or paravalvular regurgitation was evident, and no structural or non-structural valve dysfunctions and no valve thrombosis were observed before discharge [10].

Our study showed similar early clinical results on TTE after implantation of the bioprosthetic valve that were within normal limits at discharge. Yet, this is the first study to report an abnormally elevated early deterioration of the Labcor Dokimos valve on long-term follow-up for up to 8 years. A large portion of patients (26%) had earlier than expected severe SVD, as early as 2 years in our cohort. As observed, the primary mode of valve failure was pure severe AR. It is unclear why the majority of cases had pure AR without clear calcification and degeneration of the leaflets but with central leaflet non-coaptation or prolapse of one of the leaflets that may be unique for the Labcor Dokimos valve.

This report is not the first on early bioprosthetic SVD. Early SVD was found in patients who underwent SAVR with a Mitroflow bioprosthesis (which has a design similar to that of the Dokimos valve with externally mounted leaflets), especially in small sizes (19 and 21 mm), with a marked increase in mortality [5]. A recent study highlighted the unexpected SVD rate with the Trifecta bioprosthesis (Abbott Laboratories, Chicago, IL, USA) (again with an externally mounted leaflet design) within 7 years, particularly in younger patients [11]. In our study, it appears that the Labcor bioprosthetic valve provides less than expected durability. It should be noted that recent studies have highlighted how different bioprosthetic designs impact the amount of mechanical wear, because mechanical stresses are distributed differently. Externally mounted leaflet valves showed superior hydrodynamic performance, but inferior mechanical durability compared to interiorly mounted leaflet valves after 600 million cycles of testing. The primary failures occurred because of significant mechanical abrasion in the commissural region, which may warrant close monitoring of externally mounted leaflet valves during long-term follow-up [12].

Risk factors previously found to be associated with bioprosthetic SVD are younger age, mitral valve position, end-stage renal disease, higher calcium-phosphorus product, hyperparathyroidism, hypertension and pregnancy. These findings support the implication of lipid-mediated inflammation in the calcific degeneration of bioprosthetic valve leaflets [13]. Prosthesis-related factors include small bioprosthesis size and PPM whereby these factors may increase mechanical stress on the leaflets [13]. PPM and small size were not found to be predictors of SVD in our report, most likely due to the small sample size.

Given that 26% of patients who were followed up developed severe SVD early after SAVR, large numbers of patients are anticipated to develop clinically relevant SVD in the near future, some requiring reintervention. Many patients present with severe valve failure due to rapid onset of valve obstruction or regurgitation, with a high risk for emergent reoperation. Emergent repeat SAVR is associated with a mortality of 22.6% compared to 1.4% for elective redo SAVR [14]. It is crucial that patients who have SAVR undergo close follow-up yearly to identify those who are at risk, thereby offering optimized timing for repeat elective interventions.

Although the reported durability of a surgical aortic bioprosthesis is >85% at 10 years, most studies to date have used reoperation instead of valve performance parameters to define valve durability, leading to a likely underestimation of the real incidence of SVD. In our report, if we take reoperation as our end-point, the rate of valve failure would be 17.6%, although the rate of severe SVD was 26% because 7 patients have not yet had the appropriate treatment.

PPM and small valves were associated with early SVD, and efforts were made to select large valve sizes or to offer TAVI with a supra-annular design to improve haemodynamics and mitigate risks of early degeneration [15]. Our study showed that a large EOA post SAVR is protective for SVD.

Moreover, the Labcor Dokimos is a challenging bioprosthesis for valve-in-valve TAVI because it is radio-opaque with no fluoroscopic landmarks, making VIV TAVI more challenging with an increased risk of coronary obstruction because it has externally mounted leaflets. We performed 9 valve-in-valve TAVI procedures with this valve, with 3 cases of intentional bioprosthetic fracture, to optimize the haemodynamics (Supplementary Material, Fig. S1).

The significant increase in the use of aortic bioprostheses in recent times will inevitably lead to rising numbers of patients diagnosed with SVD in the next decade. This fact should stimulate further research efforts in the prevention and treatment of this entity, particularly if we continue treating younger patients with biological valves.

The literature on SVD is extensive. The greatest barrier to comparing the durability of bioprostheses stems from differences in the definitions of valve deterioration. The Valve Academic Research Consortium-2 recommendations define SVD as valve-related dysfunction (mean gradient ≥ 20 mmHg, EOA ≤ 0.9–1.1cm^2^, DVI 0.35 m/s, moderate or severe prosthetic regurgitation) [16].

### Limitations

These data were collected in a single-centre setting. However, the major advantage of limiting data collection to a single centre involves the inclusion of a homogeneous patient population, adherence to a constant clinical outcome and consistent quality of echocardiographic findings. The patients had similar risk scores and age groups, making it a uniform population. Our investigation is limited by its retrospective nature and the limited sample, which reduces the impact and validity of the study, and by the fact that some patients were lost to follow-up. We should emphasize that the incidence of SVD could be underestimated, considering that only 73% of the patients were followed up. This report is an initial report that highlights the incidence of SVD, but further investigations are required to study the predictors of early SVD.

## CONCLUSION

The Labcor Dokimos bioprosthetic aortic valve seems to provide less than expected durability compared with other valves. Given that moderate and severe SVD developed in 38% of patients early after SAVR, large numbers of patients are anticipated to sustain clinically relevant SVD in the near future, which may compromise the prognosis of these patients. Considering the large numbers of this valve implanted worldwide, further investigation involving other institutions using standardized methods and diagnostic criteria is highly warranted.

## IMPACT ON DAILY PRACTICE

Early SVD has a strong impact on patient outcome, with a reduced overall survival rate. Our findings advocate for yearly echocardiography from the first year after Labcor Dokimos implantation and careful monitoring of gradients and aortic regurgitation.

## SUPPLEMENTARY MATERIAL

Supplementary material is available at *ICVTS* online.

**Conflict of interest:** none declared.

### Data Availability Statement

The data underlying this article are available in the article and in its online supplementary material. Upon request, a full Excel sheet that includes demographics, procedural data, and echocardiography data can be obtained.

### Author contributions

**Dounia Iskandarani:** Conceptualization; Data curation; Formal analysis; Investigation; Methodology; Project administration; Resources; Writing—original draft; Writing—review & editing. **Omar Chaabo:** Data curation; Investigation; Resources. **Walid Gharzeddine:** Data curation; Investigation; Resources; Supervision; Validation. **Pierre Sfeir:** Conceptualization; Formal analysis; Investigation; Validation. **Mounir Obeid:** Data curation; Investigation; Resources; Supervision; Visualization. **Ziyad Ghazzal:** Conceptualization; Data curation; Investigation; Methodology; Project administration; Validation; Visualization. **Abdallah Rebeiz:** Investigation; Methodology; Resources; Supervision; Validation. **Fadi J. Sawaya:** Conceptualization; Formal analysis; Investigation; Methodology; Project administration; Resources; Supervision; Validation; Writing—review & editing.

### Reviewer information

Interactive CardioVascular and Thoracic Surgery thanks Clarence Pienteu Pingpoh, Prakash P Punjabi, Giuseppe Santarpino and the other, anonymous reviewer(s) for their contribution to the peer review process of this article.

## Supplementary Material

ivab286_Supplementary_Data

## ABBREVIATIONS

AR: Aortic regurgitation
AS: Aortic stenosis
DVI: Doppler velocity index
EOA: Effective orifice area
EOAi: Indexed effective orifice area
LVOT: Left ventricular outflow tract
PPM: Patient–prosthesis mismatch
SAVR: Surgical aortic valve replacement
SVD: Structural valve deterioration
TAVI: Transcatheter aortic valve implantation
TTE: Transthoracic echocardiography

## References

[1] Nishimura RA , Otto CM, Bonow RO, Carabello BA, Erwin JP, Guyton RA et al 2014 AHA/ACC guideline for the management of patients with valvular heart disease: a report of the American College of Cardiology/American Heart Association Task Force on Practice Guidelines. J Am Coll Cardiol 2014;63:e57-185–2488. 24603191 10.1016/j.jacc.2014.02.536

[2] Baumgartner H , Falk V, Bax JJ, De Bonis M, Hamm C, Holm PJ et al; ESC Scientific Document Group. 2017 ESC/EACTS guidelines for the management of valvular heart disease. Eur Heart J 2017;38:2739–91. 28886619 10.1093/eurheartj/ehx391

[3] Dvir D , Bourguignon T, Otto CM, Hahn RT, Rosenhek R, Webb JG et al; VIVID (Valve in Valve International Data) Investigators. Standardized definition of structural valve degeneration for surgical and transcatheter bioprosthetic aortic valves. Circulation 2018;137:388–99. 29358344 10.1161/CIRCULATIONAHA.117.030729

[4] Cartlidge TR , Doris MK, Sellers SL, Pawade TA, White AC, Pessotto R et al Detection and prediction of bioprosthetic aortic valve degeneration. J Am Coll Cardiol 2019;73:1107–19. 30871693 10.1016/j.jacc.2018.12.056 PMC6424589

[5] Sénage T , Le Tourneau T, Foucher Y, Pattier S, Cueff C, Michel M et al Early structural valve deterioration of Mitroflow aortic bioprosthesis: mode, incidence, and impact on outcome in a large cohort of patients. Circulation 2014;130:2012–20. 25355912 10.1161/CIRCULATIONAHA.114.010400

[6] Labcor. DOKIMOS PLUS—Stented Pericardial Heart Valve. http://www.labcor.com.br/produtos-detalhes.php?lang=en&pag=&uid=64.

[7] Head S , Mokhles M, Osnabrugge RL, Pibarot P, Mack MJ, Takkenberg JJ et al The impact of prosthesis-patient mismatch on long-term survival after aortic valve replacement: a systematic review and meta-analysis of 34 observational studies comprising 27,186 patients with 133,141 patient-years. Eur Heart J 2012;33:1518–29. 22408037 10.1093/eurheartj/ehs003

[8] Capodanno D , Petronio AS, Prendergast B, Eltchaninoff H, Vahanian A, Modine T et al Standardized definitions of structural deterioration and valve failure in assessing long-term durability of transcatheter and surgical aortic bioprosthetic valves: a consensus statement from the European Association of Percutaneous Cardiovascular Interventions (EAPCI) endorsed by the European Society of Cardiology (ESC) and the European Association for Cardio-Thoracic Surgery (EACTS). Eur Heart J 2017;38:3382–90. 29020344 10.1093/eurheartj/ehx303

[9] Zayat R , Arias-Pinilla J, Aljalloud A, Musetti G, Goetzenich A, Autschbach R et al Performance of the Labcor Dokimos Plus pericardial aortic prosthesis: a single-centre experience. Interact CardioVasc Thorac Surg 2017;24:355–62. 28025312 10.1093/icvts/ivw401

[10] Christ T , Zhigalov K, Konertz W, Holinski S. Clinical outcome and hemodynamic behavior of the Labcor Dokimos Plus aortic valve. J Cardiothorac Surg 2016;11:160. 27899119 10.1186/s13019-016-0561-5 PMC5129248

[11] Fukuhara S , Shiomi S, Yang B, Kim K, Bolling SF, Haft J et al Early structural valve degeneration of Trifecta bioprosthesis. Ann Thorac Surg 2020;109:720–7. 31398357 10.1016/j.athoracsur.2019.06.032

[12] Vriesendorp MD , de Lind van Wijngaarden RAF, Rao V, Moront MG, Patel HJ, Sarnowski E et al An *in vitro* comparison of internally versus externally mounted leaflets in surgical aortic bioprostheses. Interact CardioVasc Thorac Surg 2020;30:417–23.423. 31778161 10.1093/icvts/ivz277

[13] Côté N , Pibarot P, Clavel MA. Incidence, risk factors, clinical impact, and management of bioprosthesis structural valve degeneration. Curr Opin Cardiol 2017;32:123–9. 28067715 10.1097/HCO.0000000000000372

[14] Vogt PR , Brunner-LaRocca HP, Sidler P, Zünd G, Truniger K, Lachat M et al Reoperative surgery for degenerated aortic bioprostheses: predictors for emergency surgery and reoperative mortality. Eur J Cardiothorac Surg 2000;17:134–9. 10731648 10.1016/s1010-7940(99)00363-2

[15] Rodriguez-Gabella R , Voisine P, Puri R, Pibarot P, Rodes-Cabau J. Aortic bioprosthetic valve durability incidence, mechanicm, predictors and management of surgical and transcatheter valve degeneration. J Am Coll Cardiol 2017;70:1013–28. 28818190 10.1016/j.jacc.2017.07.715

[16] Kappetein AP , Head SJ, Genereux P, Piazza N, van Mieghem NM, Blackstone EH et al Updated standardized endpoint definitions for transcatheter aortic valve implantation: the Valve Academic Research Consortium-2 Consensus Document. J Am Coll Cardiol 2012;60:1438–54. 23036636 10.1016/j.jacc.2012.09.001

