# Supplementary figures and images for: Structural valve deterioration of the Labcor Dokimos aortic prosthesis: a single-centre experience
Source: Interact Cardiovasc Thorac Surg. 2021 Oct 23;34(6):966–73. doi: 10.1093/icvts/ivab286 (PMC10634400; doi:10.1093/icvts/ivab286)

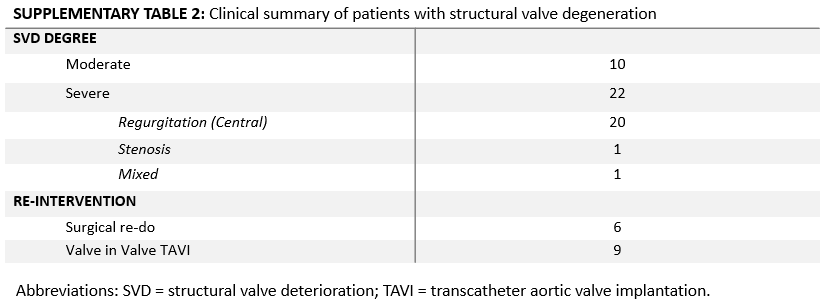

Supplement: ivab286_Supplementary_Data [file ivab286_supplementary_data.zip › ivab286-suppl_data/SupplementaryTable2.png]

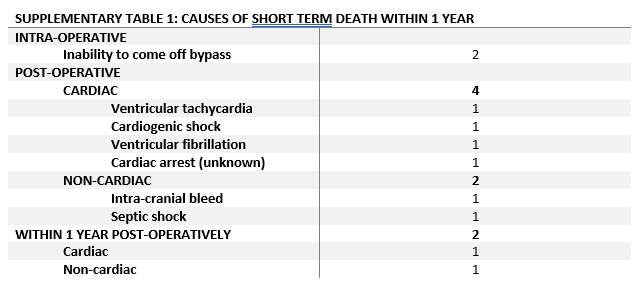

Supplement: ivab286_Supplementary_Data [file ivab286_supplementary_data.zip › ivab286-suppl_data/Supplmentary Table 1.png]

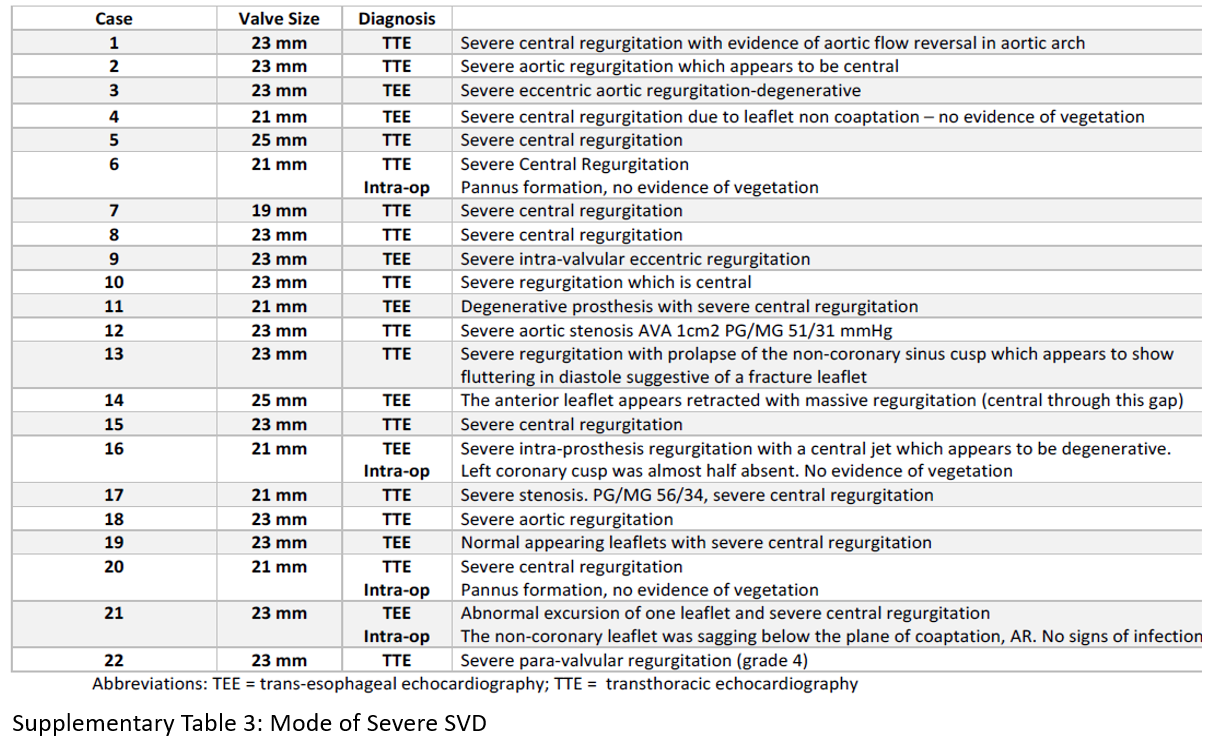

Supplement: ivab286_Supplementary_Data [file ivab286_supplementary_data.zip › ivab286-suppl_data/SuppTable3.png]
